# Prevalence and risk factors of intestinal parasitism among two indigenous sub-ethnic groups in Peninsular Malaysia

**DOI:** 10.1186/s40249-016-0168-z

**Published:** 2016-07-18

**Authors:** Yuee Teng Chin, Yvonne Ai Lian Lim, Chun Wie Chong, Cindy Shuan Ju Teh, Ivan Kok Seng Yap, Soo Ching Lee, Mian Zi Tee, Vinnie Wei Yin Siow, Kek Heng Chua

**Affiliations:** Department of Biomedical Science, Faculty of Medicine, University of Malaya, 50603 Kuala Lumpur, Malaysia; Department of Parasitology, Faculty of Medicine, University of Malaya, 50603 Kuala Lumpur, Malaysia; Department of Life Sciences, School of Pharmacy, International Medical University, 57000 Kuala Lumpur, Malaysia; Department of Medical Microbiology, Faculty of Medicine, University of Malaya, 50603 Kuala Lumpur, Malaysia

**Keywords:** Intestinal parasites, Intestinal parasitism, Indigenous people, Risk factors, Prevalence, Sub-ethnic group, Temuan sub-ethnic group, Mah Meri sub-ethnic group, Peninsular Malaysia

## Abstract

**Background:**

Intestinal parasitic infections (IPIs) among indigenous people have been widely documented in Malaysia, however, the prevalence of these infections remains high. In the past, most studies have focused on specific species of parasites but polyparasitism has received limited attention. In addition, epidemiology studies on indigenous people tend to consider them as a homogenous group, whereas in reality different sub-ethnic groups have different cultural and living practices. Variations in living habits such as personal hygiene practices may predispose different groups to different parasitic infections. To better understand prevalence and risk factors of intestinal parasitism among different sub-ethnic groups, the present study was conducted among two sub-ethnic groups of indigenous people (Temuan and Mah Meri) residing in Selangor state, Malaysia.

**Methods:**

A cross-sectional study that focused on two distinct sub-ethnic groups was carried out from February to September 2014. Faecal samples were collected from 186 participants and examined using the formalin-ether sedimentation technique. A molecular approach was adopted to conduct a genetic characterisation of the parasites. Additionally, questionnaires were administered to obtain information on the demographics, socio-economic backgrounds and behavioural risks relating to the participants, as well as information about their environments. Statistical analyses (i.e. binary and multivariate logistic regression analyses) were performed to measure risk factors.

**Results:**

For Temuan communities, trichuriasis (64.2 %) was the most common infection found, preceding hookworm infection (34 %), ascariasis (7.5 %), giardiasis (14.2 %) and amoebiasis (7.5 %). As for the Mah Meri communities, trichuriasis (77.5 %) prevailed over ascariasis (21.3 %), hookworm (15 %), giardiasis (7.5 %) and amoebiasis (3.8 %). Significant differences in proportions of trichuriasis, ascariasis and hookworm infections were observed between the Temuan and Mah Meri sub-ethnic groups. Polyparasitism was more common among the Temuan sub-ethnic group (41.5 %) compared to the Mah Meri sub-ethnic group (32.5 %), with the majority of participants harbouring two parasites concurrently (Temuan: 33 %, Mah Meri: 20 %). *Trichuris trichiura* and *Ascaris lumbricoides* co-infections were most prevalent (10 %) among the Mah Meri communities, while a co-infection of *T. trichiura* with hookworm (19.8 %) was most common among the Temuan communities. Multivariate analyses showed that being unemployed, having a large family and drinking unboiled water were found to be significantly associated with intestinal parasitism.

**Conclusion:**

The present study highlights substantial polyparasitism and risk factors for infections in the Temuan and Mah Meri sub-ethnic groups. The high prevalence of IPIs among these two sub-ethnic groups indicates that parasitic infections are important health issues in these communities. Hence, it is imperative to implement sound intervention strategies such as periodic preventive chemotherapy coupled with health education in order to reduce and eradicate these infections.

**Electronic supplementary material:**

The online version of this article (doi:10.1186/s40249-016-0168-z) contains supplementary material, which is available to authorized users.

## Multilingual abstracts

Please see Additional file 1 for translations of the abstract into the five official working languages of the United Nations.

## Background

According to the World Health Organization, it is estimated that more than 24 % of people worldwide are infected with intestinal parasitic infections (IPIs), the majority of whom reside in developing countries [1]. These infections are amongst the most common infections worldwide, with the most endemic regions being Sub-Saharan Africa, Southeast Asia, China, South India and South America [2, 3]. Helminth parasites cause IPIs, and these include soil-transmitted helminths (STHs) (i.e. *Trichuris trichiura*, *Ascaris lumbricoides*, hookworm, *Strongyloides stercoralis*), *Taenia* spp. and enteric protozoan pathogens (i.e. *Giardia duodenalis*, *Entamoeba histolytica*, *Cryptosporidium* spp.).

Indigenous communities constitute up to 0.6 % (approximately 180 000) of Malaysia’s total population [4]. Despite rapid socio-economic development and demographic changes in Malaysia over recent decades, IPIs are still highly endemic especially among indigenous communities. Epidemiology of IPIs among indigenous populations has been extensively studied with recorded prevalence rates ranging from 52.4 to 98.4 % [5–9]. However, these statistics might be masked by the fact that the majority of these reports focused on either a few targeted species of parasites or a general cluster of helminth/protozoan infections. It is surprising that polyparasitism has received only token attention and resulted in limited research in the country, whilst studies across multiple epidemiological settings throughout the world have shown that polyparasitism is the norm rather than the exception [10, 11]. Although a recent study by Al-Delaimy et al. [5] highlighted the high incidence of polyparasitism (71.4 %) in the indigenous population, the study was limited to a single community with a narrow age range (school-aged children). Similarly, as part of a small gut microbiota survey on Malaysian children, polyparasitism was detected in all surveyed indigenous Temiar children [12].

Another limitation of the previous studies was that they mainly focused on a single indigenous sub-ethnic group or regarded the overall indigenous people as a homogenous group. Indigenous people of Peninsular Malaysia comprise 18 sub-ethnic groups, which can be classified under three main ethnolinguistic groups, namely Senoi, Proto-Malay and Negrito [13]. These sub-ethnic groups are highly diverse genetically, geographically, socio-economically and culturally [13, 14], and these heterogeneities need to be examined in order to understand the variation in the prevalence of IPIs among them. The acquired information will be beneficial for formulating mechanisms for proper disease management that caters to specific groups if required, in order to effectively alleviate the disease burden.

A number of recent studies have been carried out exploring the variation of parasitic infections in different sub-ethnic groups. For instance, Ngui et al. [9] and Anuar et al. [15] have highlighted the prevalence of parasitic infections among five sub-ethnic groups (Temuan, Mah Meri, Orang Kuala, Jakun and Semelai) and three ethnolinguistic groups. Lee et al. [13] demonstrated the prevalence of STH and protozoan co-infections among Temuan and Temiar sub-ethnic groups. However, epidemiology data from these cross-sectional studies was yielded via microscopic examination and further characterisation of parasite species was not carried out.

Conventional microscopic examination remains the gold standard for parasite quantification and identification. However, this technique is less effective in differentiating species with similar morphology [16]. Due to its higher sensitivity and specificity, a molecular approach such as the polymerase chain reaction (PCR) has been increasingly used to enhance detection and differentiation of parasites in order to provide a more accurate diagnosis.

In view of the shortcomings of the research areas highlighted above, an understanding of the prevalence (i.e. monoparasitism and polyparasitism) among different sub-ethnic groups of indigenous people is very much warranted. The data will be beneficial for planning and formulating effective control measures and strategies that cater to the needs of each sub-ethnic group. The present study was therefore carried out to determine the current status, distribution and risk factors of IPIs in two sub-ethnic groups of indigenous populations, namely Temuan and Mah Meri, from four geographically distinct locations in Peninsular Malaysia.

## Methods

### Study area

**Fig. 1:**
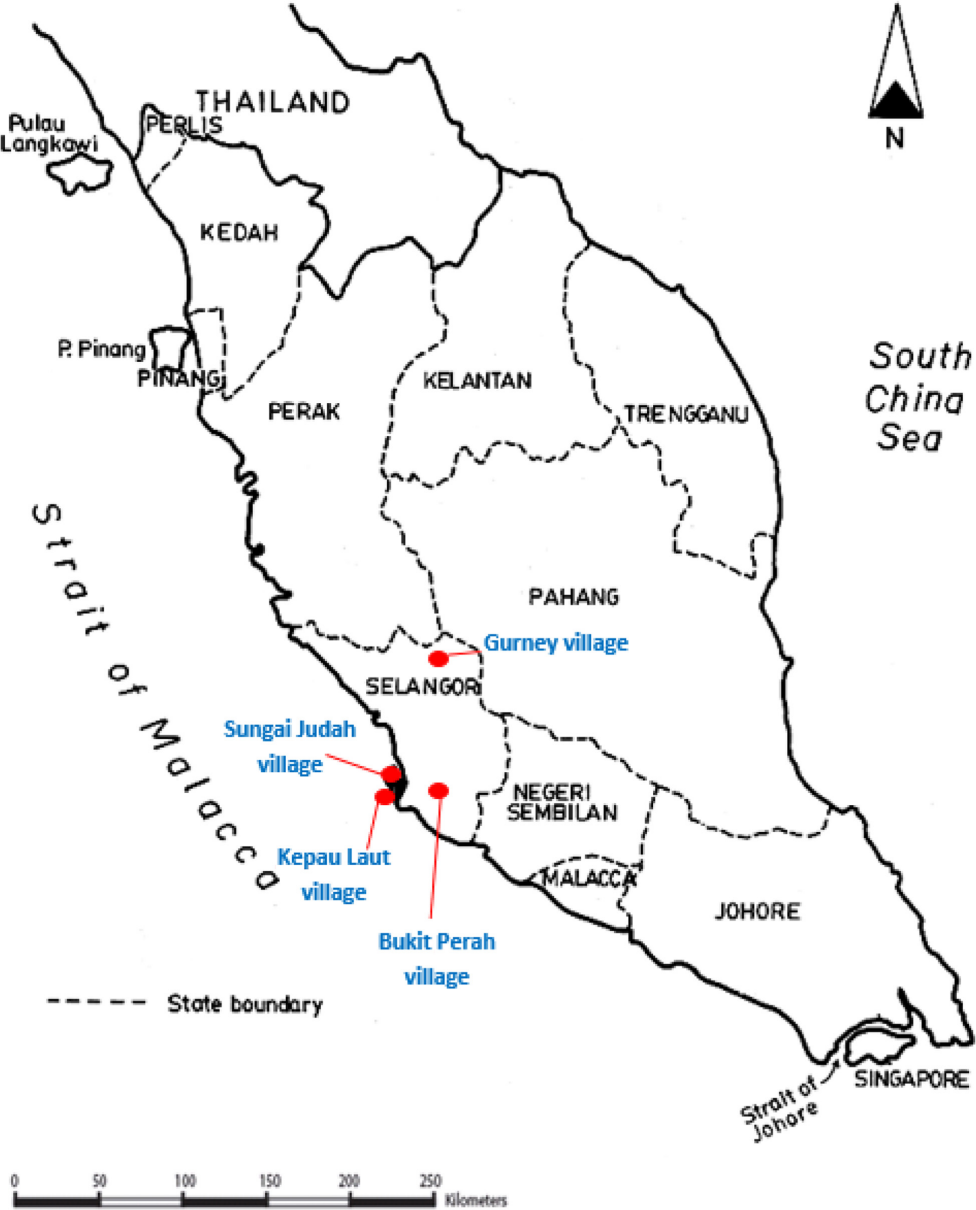
Map of sampling sites. This map shows the locations of the four sampling sites in Selangor state, Peninsular Malaysia. The sub-ethic group residing in the Gurney and Bukit Perah villages is Temuan, while the sub-ethic group residing in the Kepau Laut and Sungai Judah villages is Mah Meri

A cross-sectional study that focused on two distinct sub-ethnic groups was carried out from February to September 2014 in four different villages (i.e. Gurney, Kepau Laut, Sungai Judah and Bukit Perah) in Selangor state, Peninsular Malaysia (see Fig. 1). These four accessible villages were selected from an official list provided by the Department of Orang Asli Development (JAKOA), taking into consideration the following criteria: (1) accessibility by road and (2) willingness of villagers to participate.

Gurney village, Hulu Yam (101.67^o^ E, 3.42^o^ N) is considered a suburban area and is situated approximately 40 km from the city of Kuala Lumpur. This village is located at the fringe of a forest. Bukit Perah village, Banting (101.56^o^ E, 2.86^o^ N) is considered a suburban area with a population of approximately 200 inhabitants. The village is located at the southern part of Selangor and is surrounded by oil palm plantations. The sub-ethnic group residing in both these villages is Temuan, which belongs to the Proto-Malay ethnic group.

Kepau Laut village (101.39^o^ E, 2.84^o^ N) and Sungai Judah village (101.37^o^ E, 2.85^o^ N) are located in Carey Island, approximately 35 km from the town of Klang. Carey Island is situated on the coast of Selangor and is considered a suburban area. Both villages are surrounded by oil palm plantations. The sub-ethic group residing in these two villages is Mah Meri, which belongs to the Senoi ethnic group.

### Sample size

The sample size was calculated according to the latest study on the prevalence of IPIs among indigenous communities in Malaysia – the prevalence of 98.4 % was considered as per the study by Al-Delaimy et al. [5]. The sample size was calculated using the formula by Leedy et al. [17], taking into consideration a 95 % confidence level, with 5 % bound on the error of estimation. The minimum sample size required was 24 participants. A simple random sampling method was used in the current study, in which 186 samples were successfully collected from consenting participants.

### Structured questionnaire and sample collection

A structured questionnaire was written in English and translated into the Malay language, which is the national language of Malaysia (Bahasa Malaysia). Bahasa Malaysia is the common second language of the indigenous populations, as it is the main language used by villagers to communicate with ‘outsiders’. In all four locations, the villagers have a good command of the national language. The participants were interviewed by trained field assistants who recorded the information pertinent to the participants’ demographics (i.e., age, gender, educational attainment); socio-economic backgrounds (i.e., occupation, household income); behavioural risks (i.e., personal hygiene practices such as hand washing, defecation habits, use of footwear); medical treatments (i.e. whether the participant had taken any anthelmintic drugs); and environmental sanitation conditions (i.e. latrine systems, types of water supply, garbage disposal systems). Children who were under 12 years of age had their questionnaires filled out by their parents/guardians being interviewed, who also gave informed consent on their behalf.

After completing the questionnaire, a clean, wide-mouth screw-cap container pre-labelled with the individual’s name and code was given to each participant for the collection of a faecal sample. Participants were guided on the proper methods of providing faecal samples. Containers with the individual’s faecal samples were collected on the subsequent day.

### Faecal examination

The fresh faecal samples were transported back to the Department of Parasitology, Faculty of Medicine, University of Malaya, on the same day of collection. Faecal samples were then preserved in 2.5 % potassium dichromate and kept at 4 °C until further analysis.

For the microscopic examination of faecal samples, the formalin-ether concentration technique was adopted. Approximately 1 g to 2 g of faecal samples was mixed with 7 ml of 10 % formalin and ethyl acetate in a 15 ml Falcon® tube (Axygen® Scientific, California, USA). The samples were then centrifuged at 2500 rpm for 10 min and the supernatant was then discarded. Next, a faecal smear was made from the sediment, stained with 0.85 % iodine and examined by a light microscope under 100× and 400× magnifications. The sample was reported as microscopically positive for parasitic infection if ova of helminths or (oo)cysts of protozoa were detected.

### Molecular characterisation of parasites

Molecular characterisation of parasites was carried out for all samples using relevant published PCR protocols [18–20]. In brief, molecular identification and differentiation of hookworm species was performed using a two-step semi-nested PCR method involving the internal transcribed spacer two region and 28S ribosomal RNA region of *Necator americanus* and *Ancylostoma* spp. The PCR was performed as described in Ngui et al. [18]. As for *Entamoeba* spp., nested PCR targeting the 16S-like ribosomal RNA gene was conducted to genetically differentiate *E. histolytica*, *E. dispar* and *E. moshkovskii*. The PCR was conducted according to condition described in Ngui et al. [19]. Subsequently, identification of *Giardia* sp. based on a partial tpi gene (530 bps) was carried out using the nested PCR method, as described by Sulaiman et al. [20].

After PCR amplification, all PCR products were subjected to electrophoresis in 2 % (w/v) agarose gel and stained with SYBR® Safe DNA Gel Stain (Invitrogen, Carlsbad, California, USA). All successfully amplified PCR products were subjected to bidirectional DNA sequencing. The generated sequences were aligned and a consensus sequence was created for each sample using the BioEdit Sequence Alignment Editor program (Tom Hall Ibis Biosciences, Carlsbad, California, USA). Subsequently, similarity searches were performed using the Basic Local Alignment Search Tool. The National Centre for Biotechnology Information Reference Sequence Database was used to determine the parasite species.

### Statistical analysis

Statistical analyses were performed using the Statistical Package for the Social Sciences software for Windows (version 21, Chicago, IL, USA). Only participants who had completed entire questionnaires and had results of both the microscopic examination and molecular confirmation of parasitic infections were included in the final data analyses. For descriptive analysis, the general characteristics of the studied population, prevalence of infections and other parameters were expressed as percentages. The Pearson’s chi-square test was used to investigate the association between parasitic infections as the dependent variable, and demographic factors, socio-economic factors and personal hygiene practices as the independent variables. Parameters with *P*-values < 0.25 in the chi-square analysis were included in the logistic regression analysis. The parsimonious model was selected based on the forward likelihood ratio (*LR*). Odds ratios (*OR*s), 95 % confidence intervals (*CI*s) and *p*-values of all selected risk factors were calculated. The level of statistical significance was set at α < 0.05. The parsimonious model was built to identify the best combination of predictors for parasitic infections.

## Results

### General characteristics of the study participants

In this study, two sub-ethnic groups of indigenous communities, namely the Temuan and Mah Meri communities, were enrolled. The Temuan sub-ethnic group belongs to the Proto-Malay ethnic group, while the Mah Meri sub-ethnic group belongs to the Senoi ethnic group. The Temuan sub-ethnic group forms the largest proportion of the Proto-Malay ethnic group, with this group widely distributed in several states in Peninsular Malaysia, namely Selangor, Negeri Sembilan, Perak, Malacca and Pahang. In contrast, the Mah Meri sub-ethnic group has a population size of approximately 3 000, which is confined to the coastal area of Selangor (i.e. Carey Island).

In total, 186 participants (42.5 % males, 57.5 % females) ranging from two to 78 years of age, with a median age of 26 years (interquartile range, IQR = 12–36), participated in the current study. Among the study population, 106 participants belonged to Temuan sub-ethnic group and the remaining 80 were from the Mah Meri sub-ethnic group. For the Temuan communities, the participants ranged from two to 60 years of age with a median age of 20 years (IQR = 11–34), while the Mah Meri participants ranged from six to 78 years of age, with a median age of 30 years (IQR = 14–42).

With regards to education, 81.7 % of the total participants had formal education, with a higher percentage of Mah Meri participants (87.5 %) being educated in comparison to the Temuan participants (77.4 %). Nevertheless, poverty is still rife in these two indigenous sub-ethnic groups, especially in Mah Meri communities with 87.5 % of the families having a monthly income of less than RM750, which is below the poverty threshold in Malaysia [21].

In addition, more than half of the participants from each sub-ethnic group had large families with more than five household members (Temuan, 68.9 %; Mah Meri 58.8 %). There were similar proportions of unemployment participants in both the Temuan and Mah Meri communities (71.7 % versus 68.8 %). Among the working Mah Meri participants, the majority of them were fishermen (56 %), and the rest were engaged in factory work (8 %), agriculture (4 %) and other occupations. As for the Temuan participants, 30 % of them were farmers, 6.7 % each were rubber tappers and factory workers, 3.3 % were government servants, while others were self-employed or engaged in other related occupations.

Large proportions of Temuan and Mah Meri homes were connected to treated water supplies, which met the National Drinking Water Quality Standard [22] (Temuan: 71.7 %, Mah Meri: 78.7 %), and equipped with functional toilets (Temuan: 80.2 %, Mah Meri: 82.5 %). They also kept domestic animals (Temuan: 65.1 %, Mah Meri: 55 %), and practised proper hygiene such as boiling water before consumption (Temuan: 68.9 %, Mah Meri: 66.3 %) and wearing footwear outside their homes (Temuan: 24.5 %, Mah Meri: 27.5 %). The detailed demographic and socio-economic characteristics of the participants are presented in (Additional file 2: Table S1).

### Prevalence and distribution of IPIs

Out of the Temuan participants, 76.4 % were infected with intestinal parasites, with *T. trichiura* being the most common parasite (64.2 %), preceding hookworm (34.0 %), *A. lumbricoides* (7.5 %), *Giardia* sp. (14.2 %) and *Entamoeba* spp. (7.5 %). Of the entire Temuan participants (*n* = 106), 34.9 % were infected with a single parasite, while 41.5 % had polyparasitism. With regards to polyparasitism, 33 % harboured two different parasite species concurrently, in which co-infection of *T. trichiura* with hookworm was the most prevalent (19.8 %), followed by infections of *T. trichiura* with *Giardia* sp. (5.7 %). Among the 7.5 % of participants who had triple infections, half were co-infected with *T. trichiura*, hookworm and *Giardia* sp. Moreover, one individual was found to be concurrently infected with four types of parasites.

As for the Mah Meri sub-ethnic group, 80 % of participants had parasitic infections, with trichuriasis being the most prevalent infection (77.5 %) compared to the other IPIs. However, a slightly different trend was observed among the Mah Meri sub-ethnic group: the prevalence rate of ascariasis (21.3 %) was higher than hookworm (15.0 %) in comparison to the Temuan sub-ethnic group. In contrast to the Temuan sub-ethnic group, monoparasitism (47.5 %) was more common than polyparasitism (32.5 %) in the Mah Meri communities. Additionally, co-infection of *T. trichiura* with *A. lumbricoides* (10.0 %) was the predominant co-infection among the Mah Meri participants. Among the 12.5 % of participants who had triple infections, *T. trichiura*, *A. lumbricoides* and hookworm co-infections were the most prevalent (6.3 %).

Based on the types of IPIs, a statistical analysis was performed, which indicated significant differences of disease proportions (*P* < 0.05), namely trichuriasis, ascariasis and hookworm infection, between the Temuan and Mah Meri sub-ethnic groups. However, no significant differences in disease proportions were observed for monoparasitism and polyparasitism between these communities. The prevalence and distribution of IPIs among the two groups are shown in Table 1.

**Table 1:** Prevalence and distribution of IPIs among the Temuan and Mah Meri sub-ethnic groups

IPIs	Total (*n* = 186)		Temuan (*n* = 106)		Mah Meri (*n* = 80)	
	*n* positive (%)	95 % *CI*	*n* positive (%)	95 % *CI*	*n* positive (%)	95 % *CI*
Overall IPIs	145 (78.0)	71.5–83.3	81 (76.4)	67.5–83.5	64 (80.0)	70.0–87.3
Helminth infections						
*Trichuris trichiura*	130 (69.9)*	63.0–76.0	68 (64.2)	54.7–72.6	62 (77.5)	67.2–85.3
*Ascaris lumbricoides*	25 (13.4)*	10.2–23.6	8 (7.5)	3.9–14.2	17 (21.3)	13.7–31.4
Hookworm	48 (25.8)*	20.1–32.5	36 (34.0)	25.7–43.4	12 (15.0)	8.8–24.4
Protozoan infections						
*Giardia* sp.	21 (11.3)	7.5–16.6	15 (14.2)	8.8–22.0	6 (7.5)	3.5–15.4
*Entamoeba* spp.	11 (5.9)	3.3–10.3	8 (7.5)	3.9–14.2	3 (3.8)	1.3–10.5
Types of parasitism						
Monoparasitism						
*Trichuris trichiura*	62 (33.3)	30.0–40.1	26 (24.5)	17.3–33.5	36 (45.0)	34.6–55.9
*Ascaris lumbricoides*	1 (0.5)	0.1–3.0	–	–	1 (1.3)	0.2–6.8
Hookworm	7 (3.8)	1.8–7.6	6 (5.7)	2.6–11.8	1 (1.3)	0.2–6.8
*Giardia duodenalis*	2 (1.1)	0.3–3.8	2 (1.9)	0.5-6.6	–	–
*Entamoeba* spp.	3 (1.6)	0.6–4.6	3 (2.8)	1.0–8.0	–	–
Total single parasitic infections	75 (40.3)	33.5–47.5	37 (34.9)	26.5–44.4	38 (47.5)	36.9–58.3
Polyparasitism						
Two parasites						
*T. trichiura* and *A. lumbricoides*	11 (5.9)	3.3–10.3	3 (2.8)	1.0–8.0	8 (10.0)	5.2–18.5
*T. trichiura* and hookworm	25 (13.4)	9.3–19.1	21 (19.8)	13.3–28.4	4 (5.0)	2.0–12.2
*T. trichiura* and *Entamoeba* spp.	5 (2.7)	1.2–6.1	3 (2.8)	1.0–8.0	2 (2.5)	0.7–8.7
*T. trichiura* and *Giardia* sp.	8 (4.3)	2.2–8.3	6 (5.7)	2.6–11.8	2 (2.5)	0.7–8.7
*A. lumbricoides* and *Entamoeba* spp.	1 (0.5)	0.1–3.0	1 (0.9)	0.2–5.2	–	–
Hookworm and *Giardia* sp.	1 (0.5)	0.1–3.0	1 (0.9)	0.2–5.2	–	–
Total with two parasites	51 (27.4)	21.5–34.2	35 (33.0)	24.8–42.4	16 (20.0)	12.7–30.1
Three parasites						
*T. trichiura* and *A. lumbricoides* and hookworm	8 (4.3)	2.2–8.3	3 (2.8)	1.0–8.0	5 (6.3)	2.7–13.8
*T. trichiura* and *A. lumbricoides* and *Giardia* sp.	3 (1.6)	0.6–4.6	–	–	3 (3.8)	1.3–10.5
*T. trichiura* and *Entamoeba* spp. and *Giardia* sp.	1 (0.5)	0.1–3.0	1 (0.9)	0.2–5.2	–	–
*T. trichiura* and hookworm and *Giardia* sp.	5 (2.7)	1.2–6.1	4 (3.8)	1.5–9.3	1 (1.3)	0.2–6.8
*T. trichiura* and hookworm and *Entamoeba* spp.	1 (0.5)	0.1–3.0	–	–	1 (1.3)	0.2–6.8
Total with three parasites	18 (9.7)	6.2–14.8	8 (7.5)	3.9–14.2	10 (12.5)	6.9–21.5
Four parasites						
*T. trichiura* and *A. lumbricoides* and hookworm and *Giardia* sp.	1 (0.5)	0.1–3.0	1 (0.9)	0.2–5.2	–	–
Total with four parasites	1 (0.5)	0.1–3.0	1 (0.9)	0.2–5.2	–	–
Overall polyparasitism	70 (37.6)	31.0–44.8	44 (41.5)	32.6–51.0	26 (32.5)	23.2–43.4

*Indicates significant differences in disease proportion (*P* < 0.05) between the Temuan and Mah Meri sub-ethnic groups

### Molecular characterisation of parasitic infections

Molecular detection and characterisation of intestinal parasites namely hookworm, *Entamoeba* spp. and *Giardia* sp. were conducted for all faecal samples including samples that tested negative for infection using microscopic examination (see Table 2).

**Table 2:** Molecular characterisation via PCR and distribution of parasites among the Temuan and Mah Meri sub-ethnic groups

Intestinal parasites	Total (*n* = 186)		Temuan (*n* = 106)		Mah Meri (*n* = 80)	
	*n* positive	%	*n* positive	%	*n* positive	%
Hookworm						
*Necator americanus*	38	20.4	29	27.4	9	11.3
*Ancylostoma ceylanicum*	8	4.3	5	4.7	3	3.8
*N. americanus* and *A. ceylanicum*	2	1.1	2	1.9	0	0
*Entamoeba* spp.						
*Entamoeba histolytica*	7	3.8	5	4.7	2	2.5
*Entamoeba dispar*	2	1.1	1	0.9	1	1.3
*E. histolytica* and *E. dispar*	2	1.1	2	1.9	0	0
*Giardia* sp.						
*Giardia duodenalis*	21	11.3	15	14.2	6	7.5

For molecular characterisation of hookworm, two types of hookworm species were found in hookworm positive faecal samples, namely *N. americanus* and *A. ceylanicum*. Among the Temuan communities, a significant proportion of participants (27.4 %) was infected with *N. americanus*, but only 4.7 % harboured the *A. ceylanicum* parasite. Moreover, mixed infections of *N. americanus* with *A. ceylanicum* were also observed among Temuan participants with a prevalence rate of 1.9 %. As for the Mah Meri communities, single hookworm infections were found, with prevalence rates of 11.3 % for *N. americanus* and 3.8 % for *A. ceylanicum*.

With regards to protozoan infections, *Giardia duodenalis* was the most prevalent protozoa found in both Temuan and Mah Meri sub-ethnic groups, with prevalence rates of 14.2 and 7.5 %, respectively. For amoebiasis, two types of *Entamoeba* spp. including *E. histolytica* and *E. dispar* were detected. The former was the predominant species in both communities: 4.7 % in the Temuan participants and 2.5 % in the Mah Meri participants. In contrast, only one individual each from the two sub-ethnic groups harboured *E. dispar*. Additionally, co-infection of *E. histolytica* with *E. dispar* was reported among 1.9 % of the Temuan communities, but no such case was found in the Mah Meri communities.

### Risk factors associated with IPIs

Table 3 shows the results of the univariate analysis, which determined the association between IPIs, and participants’ demographic and socio-economic factors, personal hygiene practices and environmental factors. Table 4 further summarises the significant risk factors for infections as determined by the multivariate logistic regression analysis.

**Table 3:** Univariate analysis of factors associated with IPIs among the Temuan and Mah Meri sub-ethnic groups

Risk factors	Total (*n* = 186)				Temuan (*n* = 106)				Mah Meri (*n* = 80)			
	No. examined	Infected, *n*(%)	*OR* (95 % *CI*)	*P-*value	No. examined	Infected, *n*(%)	*OR* (95 % *CI*)	*P-*Value	No. examined	Infected, *n*(%)	*OR* (95 % *CI*)	*P*-Value
Demographic factors												
Sub-ethnic group												
Temuan	106	81 (76.4)	1.24 (0.61–2.51)	0.559	N/A	N/A	N/A	N/A	N/A	N/A	N/A	N/A
Mah Meri	80	64 (80.0)	1		N/A	N/A	N/A		N/A	N/A	N/A	N/A
Age												
< 15 years	63	52 (82.5)	0.66 (0.30–1.42)	0.281	43	34 (79.1)	0.78 (0.31–1.97)	0.595	20	18 (90.0)	0.37 (0.08–1.77)	0.197
≥ 15 years	123	93 (75.6)	1		63	47 (74.6)	1		60	46 (76.7)	1	
Sex												
Female	107	84 (78.5)	1.08 (0.54–2.17)		63	47 (74.6)	0.78 (0.31–1.97)		44	37 (84.1)	1.76 (0.58–5.32)	
Male	79	61 (77.2)	1	0.834	43	34 (79.1)	1	0.595	36	27 (75.0)	1	0.312
Family size												
≥ 5 members	120	98 (81.7)	1.80 (0.89–3.65)		73	62 (84.9)	4.15 (1.62–10.66)		47	36 (76.6)	0.58 (0.18–1.88)	
< 5 members	66	47 (71.2)	1	0.100	33	19 (57.6)	1	0.002	33	28 (84.4)	1	0.364
Socio-economic factors												
Education attainment												
Informal education	34	27 (79.4)	1.11 (0.45–2.77)	0.821	24	18 (75.0)	0.91 (0.31–2.60)	0.853	10	9 (90.0)	2.46 (0.29–20.93)	0.398
Formal education	152	118 (77.6)	1		82	63 (76.8)	1		70	55 (78.6)	1	
Monthly household income												
< RM750	132	108 (81.8)	2.07 (1.00–4.27)	0.047	62	50 (80.6)	1.75 (0.71–4.31)	0.223	70	58 (82.9)	3.22 (0.79–13.19)	0.091
≥ RM750	54	37 (68.5)	1		44	31 (70.5)	1		10	6 (60.0)	1	
Employment status												
Unemployed	131	106 (80.9)	1.74 (0.84–3.60)	0.133	76	58 (76.3)	0.98 (0.36–2.66)	0.969	55	48 (87.3)	3.86 (1.24–12.04)	0.016
Employed	55	39 (70.9)	1		30	23 (76.7)	1		25	16 (64.0)	1	
Presence of toilets												
No	35	33 (94.3)	5.75 (1.32–25.07)	0.010	21	19 (90.5)	3.52 (0.76–16.33)	0.090	14	14 (100.0)	N/A	N/A
Yes	151	112 (74.2)	1		85	62 (72.9)	1		66	50 (75.8)		
Water source												
Untreated water source	47	44 (93.6)	5.52 (1.62–18.83)	0.003	30	27 (90.0)	3.67 (1.01–13.34)	0.038	17	17 (100.0)	N/A	N/A
Treated water source	139	101 (72.7)	1		76	54 (71.1)	1		63	47 (74.6)		
Presence of domestic animals												
Yes	113	84 (74.3)	0.57 (0.27–1.21)	0.138	69	49 (71.0)	0.38 (0.13–1.12)	0.074	44	35 (79.5)	0.94 (0.31–2.83)	0.911
No	73	61 (83.6)	1		37	32 (86.5)	1		36	29 (80.6)	1	
Personal hygiene factors												
Boiling water before drinking												
No	60	53 (88.3)	2.80 (1.16–6.75)	0.018	33	31 (93.9)	7.13 (1.57–32.37)	0.004	27	22 (81.5)	1.15 (0.36–3.74)	0.813
Yes	126	92 (73.0)	1		73	50 (68.5)	1		53	42 (79.2)	1	
Eating with bare hands												
Yes	173	134 (77.5)	0.63 (0.13–2.94)	0.548	98	74 (75.5)	0.44 (0.05–3.76)	0.442	75	60 (80.0)	1.00 (0.10–9.61)	1.000
No	13	11 (84.6)	1		8	7 (87.5)	1		5	4 (80.0)	1	
Wearing shoes outside												
No	48	46 (95.8)	9.06 (2.10–39.15)	0.001	26	26 (100.0)	N/A	N/A	22	20 (90.9)	3.18 (0.66–15.34)	0.133
Yes	138	99 (71.7)	1		80	55 (68.8)			58	44 (75.9)	1	
Washing hands after contact with soil												
No	52	47 (90.4)	3.45 (1.27–9.37)	0.011	21	21 (100.0)	N/A	N/A	31	26 (83.9)	1.51 (0.47–4.85)	0.491
Yes	134	98 (73.1)	1		85	60 (70.5)			49	38 (77.6)	1	
Washing hands before eating												
No	49	45 (91.8)	4.16 (1.40–12.38)	0.006	20	20 (100.0)	N/A	N/A	29	25 (86.2)	1.92 (0.56–6.63)	0.295
Yes	137	100 (73.0)	1		86	61 (70.9)			51	39 (76.5)	1	
Washing hands before cooking												
No	34	30 (88.2)	2.41 (0.80–7.30)	0.110	14	14 (100.0)	N/A	N/A	20	16 (80.0)	1.00 (0.28–3.54)	1.000
Yes	152	115 (75.7)	1		92	67 (72.8)			60	48 (80.0)	1	
Washing hands after defecation												
No	60	56 (93.3)	5.82 (1.97–17.21)	0.000	27	27 (100.0)	N/A	N/A	33	29 (87.9)	2.49 (0.72–8.54)	0.140
Yes	126	89 (70.6)	1		79	54 (68.4)			47	35 (74.5)	1	
Washing hands after having contact with animals												
No	37	32 (86.5)	2.04 (0.74–5.62)	0.162	14	13 (92.9)	4.59 (0.57–36.97)	0.120	23	19 (82.6)	1.27 (0.36–4.43)	0.711
Yes	149	113 (75.8)	1		92	68 (73.9)	1		57	45 (78.9)	1	
Consumption of anthelmintic drugs in the past 12 months												
No	141	112 (79.4)	1.40 (0.65–3.05)	0.390	69	53 (76.8)	1.07 (0.42–2.72)	0.896	72	59 (81.9)	2.27 (0.58–12.86)	0.192
Yes	45	33 (73.3)	1		37	28 (75.7)	1		8	5 (62.5)	1

**Table 4:** Multivariate analysis of risk factors associated with parasitic infections among indigenous communities

Risk factors	Adjusted *OR*	95 % *CI*	*P*-value of Full model	*P*-value of Forward selection model
A) Overall				
Demographic factor				
Family size (≥5 members)	1.55	0.68–3.57	0.300	
Socio-economic factors				
Monthly household income (<RM750)	1.54	0.66–3.57	0.318	
Unemployed	1.18	0.50–2.82	0.703	
Without toilet	3.79	0.67–21.36	0.132	
Untreated water source	3.66	0.92–14.53	0.066	0.007*
Presence of domestic animals	0.97	0.38–2.48	0.955	
Personal hygiene factors				
Not boiling water before consumption	1.60	0.57–4.46	0.372	
Not wearing shoes outside of home	6.09	1.04–35.71	0.045	0.014*
Not washing hands after contact with soil	1.89	0.09–38.24	0.678	
Not washing hands before eating	1.74	0.13–23.69	0.677	
Not washing hands before cooking	0.13	0.01–2.56	0.179	
Not washing hands after defecation	9.94	1.03–95.79	0.047	0.009*
Not washing hands after contact with animals	0.25	0.02–2.58	0.243	
B) Temuan sub-ethnic group				
Demographic factor				
Family size (≥5 members)	3.44	1.21–9.80	0.021	0.012*
Socio-economic factors				
Monthly household income (<RM750)	2.02	0.67–6.06	0.211	
Without toilet	1.63	0.26–10.43	0.604	
Untreated water source	0.95	0.19–4.86	0.950	
Presence of domestic animals	0.34	0.09–1.31	0.117	
Personal hygiene factors				
Not boiling water before consumption	5.96	1.20–29.52	0.029	0.023*
Not washing hands after contact with animals	3.95	0.42–37.63	0.232	
C) Mah Meri sub-ethnic group				
Demographic factor				
Age (<15 years old)	0.87	0.13–6.03	0.887	
Socioeconomic factors				
Unemployed	3.52	0.93–13.26	0.063	0.020*
Monthly household income (<RM750)	1.31	0.22–7.94	0.769	
Personal hygiene factors				
Not wearing shoes when outside home	1.59	0.23–10.93	0.635	
Not washing hands after defecation	2.71	0.69–10.65	0.154	
Not consuming anthelmintic drugs in the past 12 months	2.23	0.29–16.88	0.439	

*Indicates significant risk factors associated with parasitic infections determined using the forward selection model

In general, eight factors were found to be significantly associated with intestinal IPIs. These factors are: monthly household income of less than RM750 (*OR* = 2.07; 95 % *CI =* 1.00–4.27), absence of toilets (*OR* = 5.75; 95 % *CI* = 1.32–25.07), usage of untreated water (*OR* = 5.52; 95 % *CI* = 1.62–18.83), not boiling water before drinking (*OR* = 2.80; 95 % *CI* = 1.16–6.75), walking barefooted outside of home (*OR* = 9.06; 95 % *CI* = 2.10–39.15), not washing hands after contact with soil (*OR* = 3.45; 95 % *CI* = 1.27–9.37), not washing hands before eating (*OR* = 4.16; 95 % *CI* = 1.4–12.38) and not washing hands after using the toilet (*OR* = 5.82; 95 % *CI* = 1.97–17.21). The multivariate analysis (full model) further confirmed that participants who do not wash their hands after defecation were 9.94 times more likely to be infected with parasitic infections. Likewise, those who walk barefoot outside the home were 6.1 times more likely to become infected with IPIs. According to the parsimonious model based on forward LR, however, utilisation of untreated water source was the major risk factor that increased the transmission of IPIs in the studied communities. (Additional file 2: Table S2) shows the predictive power of both full model and forward LRs for the current results.

Our results postulate that the prevalence of parasitic infections was significantly higher among Temuan participants with a large family size (*OR* = 4.15; 95 % *CI* = 1.62–10.66), those who use untreated water (*OR* = 3.67; 95 % *CI* = 1.01–13.34) and those who drink unboiled water (*OR* = 7.13; 95 % *CI* = 1.57–32.37), as compared to their counterparts. Based on the multivariate model, individuals with a large family were 3.4 times more likely to contract infections, while people who drink unboiled water were six times more likely to acquire infections.

Conversely, for the Mah Meri communities, unemployment rate (*OR* = 3.86; 95 % *CI* = 1.24–12.04) was the only parameter showing a significant association with IPIs. The corresponding multivariate model suggested that unemployed participants were 3.7 times more likely to acquire infections than their employed counterparts.

## Discussion

Our results showed that more than 75 % of participants were positive for infections of at least one parasite species. Overall, *T. trichiura* infection was the most common IPI among both the Temuan and Mah Meri sub-ethnic groups. The observation that trichuriasis is the predominant IPI is consistent with previous reports (infection rate ~35.7–95.6 %) [5, 8, 9, 13, 15, 23].

The major factor contributing to the high prevalence of trichuriasis in these indigenous populations may be attributed to the occurrence of superinfection, a phenomenon in which the host harbouring the parasite is re-infected with the identical parasite strain, especially when the existing STH control measures are ineffective against the infection [3]. The current treatment regime used for STH control is broad-spectrum anthelmintic drugs, namely albendazole and mebendazole [6]. However, numerous studies have indicated that a single dose (400 mg) of albendazole provided limited efficacy in the treatment of trichuriasis [6, 13, 24]. Higher cure rates against trichuriasis can be obtained with a higher drug dosage, but this may lead to the emergence of drug resistance. The occurrence of potential resistance of *T. trichiura* to anthelmintic drugs has been highlighted in two intervention studies done in Malaysia [25, 26].

In recent years it has become apparent that ascariasis is the second most common IPI affecting indigenous populations, followed by hookworm infection [8, 9, 15]. Similar trends of infections were found in the Mah Meri sub-ethnic group in the current study. However, the reverse was observed for the Temuan sub-ethnic group. In fact, the prevalence of ascariasis among the Temuan sub-ethnic group (7.5 %) was at the lower margin compared to other studies previously conducted in Temuan communities (4.7–36.9 %) [9, 13, 15, 27]. These outcomes could be attributed to the recent deworming programme in these communities, as reported by the respondents during the oral interview session. In addition, the difference might also be attributed to the variation in the larvae and egg load in the soil, which in turn correlates with soil moisture and relative atmosphere humidity [18, 28, 29]. Since the prevalence of ascariasis is relatively higher in the Mah Meri communities, we speculate that the intensity of soil contamination with the *Ascaris* parasite is higher in this settlement. However, this hypothesis needs to be further confirmed by conducting detailed soil analyses.

In the present study, very high prevalence rates of hookworm infection were recorded in both the Temuan and Mah Meri sub-ethnic groups. The occurrence rate (15 %) for the Mah Meri sub-ethnic group was about four times higher compared to the 3.7 % reported by Ngui et al. [9], whilst for the Temuan sub-ethnic group, the prevalence recorded in this study (34.0 %) was approximately eight-fold higher than the findings of Lee et al. (4.1 %) [13]. It is noteworthy that varying techniques employed in various studies may be accountable for the differences in the results. In this study, a more sensitive microscopic examination coupled with PCR techniques was used, while previous studies were concluded based solely on microscopic examination. In addition, it is worth highlighting that the majority of the infected participants were in the age group of above 15 years (data not shown) and this finding is in agreement with a previous study by Anuar et al. [15]. The high prevalence rate might be attributed to the fact that the most current hookworm deworming programmes have been targeting only school children [30]. Hence, future implementation of deworming programmes should include adults who may not have access to treatment. In addition, the use of night soil may serve as a source for transmission of parasitic infections. Among the general population in Malaysia, the utilisation of night soil as fertiliser is not common, but it is a regular practice among the indigenous communities. This is because indigenous communities cannot afford to use commercial fertilisers to fertilise their crops. As a result, this practice eventually leads to contamination of agricultural land. Given that in many indigenous populations small farming activities are usually carried out in the vicinity of their homes, this contamination can easily spread to humans [5]. The association between using night soil and the increased transmission of parasitic infections has been recorded in several studies [5, 31, 32]. In the present study, approximately 70 % of participants admitted to walking barefoot whilst conducting activities outdoors and this habit may serve as a mechanism for acquiring diseases such as hookworm infections, as the infective stage of a hookworm (i.e., larva) can penetrate through human skin.

In addition, two hookworm species, namely *N. americanus* and *A. ceylanicum*, were discovered in this study via molecular characterisation. *N. americanus* was found to be the most common hookworm species infecting both sub-ethnic groups and the findings were in concordance with previous studies [18, 33, 34]. However, the presence of *A. ceylanicum* in the current study demonstrated that zoonotic transmission of parasites may have occurred as animals, particularly dogs and cats, are natural reservoirs for this parasite [35]. In recent years, zoonotic significance of *A. ceylanicum* infections in humans have been reported in Asian countries such as India, Thailand, Malaysia, Laos and Cambodia [18, 36, 37]. In the current study, more than 50 % of participants from each sub-ethnic group kept dogs or cats as pets. The close relationship with animals, poor hygiene and negligible veterinary care contribute to higher risks of zoonotic transmission of *A. ceylanicum*. Furthermore, it was surprising that co-infections of *N. americanus* with *A. ceylanicum* were detected among the Temuan community as individuals with stable chronic infections with anthroponotic hookworm have been known to be less susceptible to another closely related species, in this case the *A. ceylanicum* hookworm [36, 37]. Nevertheless, a similar observation of co-infection with closely related parasites was noted by Ngui et al. [18]. Although the underlying cause and clinical significance of the disease remains unexplored, the occurrence of the *A. ceylanicum* infection should be a public health concern.

In Malaysia, protozoan infections have frequently been reported among indigenous communities due to poverty and lack of proper basic amenities, especially a municipal water system, in these communities. Over the past decade, the prevalence of *Giardia* infection ranged from 2.0 to 29.2 %, while *E. histolytica/dispar* infections varied from around 1.0 to 18.5 % among rural communities [6, 23]. In this study, the prevalence of both giardiasis (14.2 %) and amoebiasis (7.5 %) among the Temuan communities were approximately two folds higher in comparison with the Mah Meri communities. To our best knowledge, the protozoan parasitic infection status for the Mah Meri sub-ethnic group has not been reported before. Although the current prevalence among the Mah Meri sub-ethnic group was relatively low compared to the overall indigenous population, the presence of pathogenic *E. histolytica* and *G. duodenalis* parasites in these communities is noteworthy. From the molecular differentiation of *Entamoeba* spp., pathogenic *E. histolytica* was the most prevalent *Entamoeba* spp. found among the Temuan sub-ethnic group (i.e. 7 out of 8 *Entamoeba* spp. positive samples). *E. histolytica* and *G. duodenalis* are the prevailing human pathogens that cause diarrhoea among infected individuals [27, 38]. In the current study, the majority of Temuan communities’ homes were connected to treated water supplies, but there were still some Temuan participants who were dependent on river water for daily basic activities such as drinking, bathing and washing. Thus, the usage of untreated water could be a factor contributing to widespread infections in these communities. Moreover, defecating at the site of the stream was also a common practice in the communities, especially among children [27]. As a result, the runoff of slurries and sewage sludge into rivers eventually leads to heavy contamination of water sources with *Giardia* sp., *Entamoeba* spp. and other pathogenic parasites, viruses and bacteria [39].

The present study also investigated polyparasitism among indigenous populations, as this aspect is frequently ignored in epidemiological surveys. Most available studies routinely focused on the prevalence and burden of disease of individual parasites, but overlooked the special characteristic of polyparasitism and the interactions between concurrently present parasites [11]. Current findings showed that polyparasitism was highly prevalent among indigenous populations, with nearly half of Temuan participants (41.5 %) and 32.5 % of Mah Meri participants suffering from polyparasitism. According to previous studies, the majority of polyparasitism incidents involved double and triple infections [5, 40, 41]. In the present study, both Temuan and Mah Meri participants recorded higher prevalence rates of double infections. In general, co-infection of *T. trichiura* with *A. lumbricoides* was most prevalent (10 %) among the Mah Meri participants, with similar outcomes also reported by Al-Delaimy et al. [5]. This can be attributed to the common route of transmission (i.e. faecal-oral route), especially when people do not practice proper personal hygiene practices [42]. In contrast, co-infection of *T. trichiura* with hookworm (19.8 %) was most common among the Temuan sub-ethnic group and this can be explained by the high hookworm infection rate reported in these communities.

The prevalence rates of other combinations of parasitic infections such as STH with *G. duodenalis* or *Entamoeba* spp. were also high among the participants. Our observations are in line with a previous study by Al-Delaimy et al. [5], in which co-infection of three main STH species (*T. trichiura*, *A. lumbricoides* and hookworm) was the most common triple infection, as also seen in the Mah Meri sub-ethnic group in the current study. Interestingly, one Temuan participant harboured four parasites concurrently. These findings imply that indigenous populations’ environments are heavily contaminated with parasites, with poor knowledge, attitude and practices further aggravating the situation.

Polyparasitism is not merely a marker for poor sanitation and poverty but its prevalence rates are crucial to know, as individuals with multiple infections may suffer from multiple morbidity and increased susceptibility to other infections [5]. For instance, multiple intestinal helminth infections were found to increase the likelihood of malaria episodes and co-infection of *Plasmodium*-hookworm further exacerbates iron-deficiency anaemia [10, 43]. Besides this, competition for nutrients among intestinal parasites may also be present in individuals with polyparasitism, leading to malnutrition and irreparable damage to the host’s cognitive and physical development [40]. Jardim-Botelho et al. [44] demonstrated that children with both *A. lumbricoides* and hookworm infections performed poorer in a cognitive test, while Saldiva et al. [45] reported that children who harboured a concomitant infection with *A. lumbricoides* and *T. trichiura* were at higher risk of stunting. Since nearly half of the participants with polyparasitism in our study were of younger age (below 15 years old) (data not show), it is crucial to continue investigating incidences of polyparasitism for better disease management planning.

The current study also investigated risk factors for IPIs. A large family size was identified as the major risk factor responsible for the continuous transmission of IPIs among Temuan communities. A similar observation was previously reported by Anuar et al. [15] and Nasr et al. [8]. This factor may be attributed to overcrowded household conditions and close contact with family members, which can increase the risk of intra-family transmission. Moreover, the presence of infected family members also elevates the risk of parasitic infections due to the horizontal spread or focal transmission of disease among family members [8]. In the same vein, we also found that there was a significant association between IPIs and consumption of unboiled drinking water among Temuan communities. The association between transmission of IPIs and drinking unboiled water has been well documented, especially for *Giardia* and *Cryptosporidium* parasites [27, 46]. Moreover, outbreaks of waterborne diseases due to the drinking of contaminated water have been reported elsewhere [27].

With regards to the Mah Meri sub-ethnic group, the unemployment rate was the only significant factor found to be associated with IPIs. In the current study, the majority of Mah Meri participants were children and housewives. Children usually spend most of their time playing outside, while housewives may engage in household chores in and around the house. Since the environment of the house may be heavily contaminated with parasites, these groups are likely to have a higher chance of acquiring an infection.

Besides the factors mentioned above, inadequate knowledge and awareness of hygiene among the indigenous communities such as using untreated water, infrequent use of footwear and not washing hands especially after defecation further predisposes them to IPIs. Preventive measures such as health education campaigns for children should be implemented to ensure proper habits are instilled early. In Malaysia, a well-planned health education programme for indigenous communities is still not available, leading to a low level of knowledge and an indifferent attitude towards the prevention of parasitic infections [47]. Nevertheless, an isolated success story of reducing transmission of IPIs via a trial health education learning package has been reported by Al-Delaimy et al. [48]. Their findings are consistent with health education projects conducted in Seychelles, Peruvian Amazon and China [49–51]. The success that has been achieved from this trial and other projects could serve as a benchmark for the implementation of active health education programmes in the future.

We acknowledge some limitations of the current study. First and foremost, the prevalence of IPIs in this study was solely based on a single faecal sample examination rather than the ideal three consecutive sample collections. This was due to limited resources and cultural beliefs of some indigenous people who refused to give faecal samples. Therefore, we may have underestimated the actual prevalence due to the intermittent nature of cyst excretion in the faeces. Nevertheless, a more sensitive diagnostic protocol, which is the PCR, was conducted to increase detection limits of parasitic infections. Furthermore, the indigenous communities that we covered in the present study were mainly from suburban areas. There are many indigenous villages located in deeply remote areas that are difficult to access and they were therefore not included in our study. Moreover, collection of animal samples was not included in this study and thus we were unable to estimate the prevalence of IPIs among domestic animals. Future studies involving both animal and human samples are crucial to evaluate the zoonotic transmission of IPIs, particularly hookworm infection to humans. These findings will eventually generate a better understanding of disease transmission and prevention.

## Conclusion

In conclusion, our findings suggest that there is a substantial proportion of polyparasitism among both the Mah Meri and Temuan sub-ethnic groups. Given that the epidemiological understanding of polyparasitism is still in its infancy, it is crucial to investigate the impact of co-morbidities in these populations. Moreover, the distribution and prevalence of monoparasitism and polyparasitism across different sub-ethnic groups of indigenous populations should be taken into consideration during the planning and implementation of control measures in order to address the needs of various sub-ethnic groups. Although the current study did not show significant differences of monoparasitism and polyparasitism between the Temuan and Mah Meri sub-ethnic groups, future studies should include other sub-ethnic groups as well, as the indigenous communities of Malaysia are heterogeneous in their levels of education, lifestyles and hygiene practices. Additionally, the findings that both the Mah Meri and Temuan indigenous communities harboured high levels of overall parasitic infections highlights the urgent need for disease control measures, such as periodic chemotherapy, provision of safe water and improved sanitation. To consolidate and ascertain long-term sustainability of disease control, greater efforts are required to instil understanding of personal hygiene and health education among indigenous people in order to enhance their knowledge and awareness about the transmission and prevention of these infections.

## Abbreviations

CI, confidence interval; IPI, intestinal parasitic infection; IQR, interquartile range; JAKOA, Department of Orang Asli Development; LR, likelihood ratio; OR, odds ratio; PCR, polymerase chain reaction; STH, soil-transmitted helminth.

## Additional files

Additional file 1: Multilingual abstract in the five official working languages of the United Nations. (PDF 464 kb) Additional file 2: Additional tables. Table S1. General characteristics of indigenous communities who participated in this study. **Table S2.** Predictive power of the enter and forward selection logistic regression model. (PDF 175 kb)
