# Supplementary material for: Prevalence and risk factors of intestinal parasitism among two indigenous sub-ethnic groups in Peninsular Malaysia
Source: Infect Dis Poverty. 2016 Jul 18;5:77. doi: 10.1186/s40249-016-0168-z (PMC4950084; doi:10.1186/s40249-016-0168-z)
Supplement: Additional file 1: — Multilingual abstract in the five official working languages of the United Nations. (PDF 464 kb) [file 40249_2016_168_MOESM1_ESM.pdf]

Translation of the abstract into the five official working languages of the United Nations

الفوائد الصحية والاقتصادية للبرنامج العالمي للقضاء على داء الخيطيات اللمفاوية (2000 – 2014)

هوغو جيم تيرنر، أليسون A بيتيس، وريان ك تشو، ديبورا A مكفارلاند، باميلا ي هوبر، إريك أ أوتيسين ومارك ح برادلي

موجز

نبذة: الخيطيات اللمفاوية الخلفية(LF) :،تعرف أيضا باسم داء الفيل، و هي مرض مداري مهمل (NTD) مستهدف بغرض القضاء عليه من خلال البرنامج العالمي للقضاء على مرض الخيطيات اللمفاوية الخلفية (جبيلف). بين عام 2000 وعام 2014، قدم البرنامج العالمي 5.6 بليون علاجاً إلى أكثر من 763 مليون شخص. من الضروري تحديث تقديرات الفوائد الصحية والاقتصادية لهذا الإنجاز الضخم بغرض تبرير الموارد والاستثمارات اللازمة للقضاء على هذا المرض.

الأسلوب: قمنا بالجمع بين النماذج المحددة سابقا لتقدير عدد المظاهر السريرية وسنوات العمر المعدلة حسب الإعاقة (المصححة) مستبعدة من ثلاثة مجموعات (تلك المحمية من التقاط العدوى والمصابين بالمرض تحت اكلينيكيا حالت دون إحراز تقدم والمصابين بالأعراض السريرية المخففة). تم تحليل الوفورات الاقتصادية المرتبطة بالوقاية من هذا المرض في سياق النفقات الطبية التي تكبدها المرضى السريريون، أثر فقدان الدخل المحتملة من خلال تكاليف العمل المفقودة ومنعت النظام الصحي للعناية بالأفراد. وتم احتساب تقديرات التكاليف غير المباشرة باستخدام نهج رأس المال البشري. واستخدمت مجموعة من أربع مصادر للأجور بغرض تقدير القيمة السوقية العادلة لوقت العامل الزراعي المصاب بعدوى) LF لضمان الحصول على تقديرات متحفظة باستخدام معدلات الأجور الدنيا.

النتائج: توصلنا إلى توقعات بأنه بسبب السنوات ال 15 الأولى فمن المحتمل وقاية عدد 36 مليون حالة سريرية و175 (116-250) (مصححة) من المرض من خلال البرنامج العالمي. ويقدر أنه بفضل هذه التأثيرات الصحية الملحوظة، فمن الممكن توفير مبلغ 100.5 مليون دولار أمريكي على مدى عمر المجموعات المستفيدة من البرنامج. المجموع الإجمالي النتائج من مجموع النفقات الطبية التي تكبدها المصابون بالمرض (3 مليار دولار أمريكي)، تقديرات خسارة الدخل المحتملة (مبلغ 94 مليار دولار أمريكي)، وتبلغ تكلفته على النظام الصحي (مبلغ 3.5 مليار دولار أمريكي) من المتوقع إمكان توفيرها. خضعت النتائج إلى تحليل الحساسية وكانت أكثر حساسية للنسبة المئوية المفترضة لساعات العمل المفقودة لأولئك الذين يعانون من المرض المزمن (تغيير الفائدة الاقتصادية الكلية بين 69.30 مليار – 150.7 مليار دولار أمريكي)

الاستنتاج: رغم محدودية أي تحليل من هذا القبيل، إلا أن هذه الدراسة تحدد الفوائد الصحية والاقتصادية الجمّة التي نجمت عن السنوات الخمس عشرة الأولى من تطبيق البرنامج العالمي، كما أنها تسلط الضوء على قيمة وأهمية مواصلة الاستثمار في البرنامج العالمي للقضاء على داء الخيطيات اللمفاوية

Translated from English version into Arabic by Lamya, through

TRANSLATORS
WITHOUT BORDERS

马来西亚半岛两个土著族群的肠道寄生虫感染的流行情况及危险因素

Yuee Teng Chin, Yvonne Ai Lian Lim, Chun Wie Chong, Cindy Shuan Ju Teh, Ivan Kok Seng Yap, Soo Ching Lee, Mian Zi Tee, Vinnie Wei Yin Siow and Kek Heng Chua

摘要

引言：土著人群的肠道寄生虫感染在马来西亚曾有广泛记载，而且流行水平仍然较高。过去的研究大多集中于特定的虫种，而很少关注多重感染。此外，关注土著人群的流行病学研究一般都将他们作为一个同质的人群，而事实上不同族群具有不同的文化和生活习惯。诸如个人卫生等生活习惯的差异可使不同人群感染寄生虫的风险不同。为更好地了解这些族群的肠道寄生虫流行情况和危险因素，在马来西亚 Selangor 州的 2 个土著族群（Temuan 和 Mah Meri）开展了本研究。

方法：2014 年 2-9 月在 2 个不同族群开展了一项横断面调查。收集了 186 名参与者的粪便标本并采用福尔马林-乙醚沉淀法进行检测。采用分子生物学技术检测了虫种的遗传特征。此外，通过问卷调查收集了参与者的人口学、社会经济学和行为危险因素等情况及相关环境信息。采用二变量和多变量 Logistic 回归分析测量危险因素。

结果：在 Temuan 地区，鞭虫、钩虫、蛔虫、贾第虫和阿米巴感染率分别为 64.2%、34%、7.5%、14.2%和 7.5%。在 Mah Meri 地区，鞭虫、蛔虫、钩虫、贾第虫和阿米巴分别为 77.5%、21.3%、15%、7.5%和 3.8%。两个地区的鞭虫、蛔虫和钩虫感染差异有统计学意义。多重感染在 Temuan 地区更高，构成比达 41.5%，而 Mah Meri 地区为 32.5%。其中大部分是双重感染，Temuan 地区为 33%，Mah Meri 地区为 20%。在 Mah Meri 鞭虫和蛔虫共感染最常见（10%），而在 Temuan 鞭虫和钩虫共感染最常见（19.8%）。多因素分析表明失业、家庭人数多和喝生水是感染肠道寄生虫的危险因素。

结论：本研究揭示了 Temuan 和 Mah Meri 两个少数族群肠道寄生虫多重感染情况和危险因素。以上地区肠道寄生虫感染的高流行表明寄生虫感染是该地区的重要卫生问题。因此，需要开展有力的干预措施如周期性预防性化疗和健康教育以降低及消除寄生虫病。

Translated from English version into Chinese by Men-Bao Qian, edited by edited by Pin Yang, through

CHINESE CENTER FOR DISEASE CONTROL AND PREVENTION
NATIONAL INSTITUTE OF PARASITIC DISEASES

Prévalence et facteurs de risque des parasitoses intestinales chez deux sous-groupes ethniques indigènes de la Péninsule Malaise

Yuee Teng Chin, Yvonne Ai Lian Lim, Chun Wie Chong, Cindy Shuan Ju Teh, Ivan Kok Seng Yap, Soo Ching Lee, Mian Zi Tee, Vinnie Wei Yin Siow et Kek Heng Chua

Résumé

Contexte : Alors que les parasitoses intestinales affectant les peuples indigènes sont largement documentées en Malaisie, leur prévalence reste élevée. La plupart des études réalisées par le passé se concentraient sur des espèces précises de parasites mais négligeaient pour la plupart les polyparasitoses. En outre, les études épidémiologiques des peuples autochtones tendent à considérer ceux-ci comme un groupe homogène, alors que les divers sous-groupes ethniques ont des pratiques culturelles et des modes de vie différents. Or les variations des habitudes de vie et d'hygiène peuvent entraîner des prédispositions à l'infestation par des espèces parasites différentes. Afin de mieux comprendre la prévalence et les facteurs de risque de parasitose intestinale chez différents sous-groupes ethniques, la présente étude a été menée dans deux groupes indigènes, les Temuan et les Mah Meri, vivant dans l'état de Selangor en Malaisie.

Méthodes : Une étude transversale concentrée sur deux sous-groupes ethniques distincts a été menée entre février et septembre 2014. Des échantillons de selles de 184 participants ont été recueillis et examinés par la technique de sédimentation au formol-éther. Une caractérisation génétique des parasites a été effectuée par la méthode moléculaire. En outre, des questionnaires ont été administrés afin de recueillir des informations sur la démographie, le contexte socioéconomique et les risques comportementaux des participants et sur leur environnement. Des analyses statistiques (analyses de régression logistique binaires et multivariées) ont été réalisées pour mesurer les facteurs de risque.

Résultats : Dans les communautés Temuan, la trichurose s'est avérée la parasitose la plus fréquente (64,2 %), devant l'ankylostomiase (34 %), l'ascaridiose (7,5 %), la giardiase (14,2 %) et l'amibiase (7,5 %). Dans les communautés Mah Meri, c'est encore la trichurose (77,5 %) qui prévalait sur l'ascaridiose (21,3 %), l'ankylostomiase (15 %), la giardiase (7,5 %) et l'amibiase (3,8 %). Des différences significatives dans les proportions de la trichurose, de l'ascaridiose et de l'ankylostomiase ont été observées entre les sous-groupes ethniques Temuan et Mah Meri. Les polyparasitoses étaient plus fréquentes parmi les Temuan (41,5 %) que parmi les Mah Meri (32,5 %), avec dans la plupart des cas la coexistence de deux parasites (Temuan : 33 %, Mah Meri : 20 %). Les co-infestations par *Trichuris trichiura* et *Ascaris lumbricoides* étaient les plus prévalentes (10 %) chez les Mah Meri et celles par *T. trichiura* et l'ankylostomiase (19,8 %) parmi les communautés Temuan. Les analyses multivariées ont montré que les faits d'être sans emploi, d'avoir une famille nombreuse et de boire de l'eau non bouillie étaient associés de façon significative aux parasitoses intestinales.

Conclusion : La présente étude met en lumière l'importance des polyparasitoses et les facteurs de risque d'infestation dans les sous-groupes ethniques Temuan et Mah Meri. La prévalence élevée des parasitoses intestinales dans ces sous-groupes ethniques indique qu'il s'agit là d'un problème important de santé dans ces communautés. Il est donc impératif de mettre en place des stratégies d'intervention bien pensées, par exemple une chimiothérapie préventive périodique associée à une éducation à la santé visant à réduire, voire à éradiquer ces parasitoses.

Translated from English version into French by Suzanne Assenat, through

TRANSLATORS
WITHOUT BORDERS

Распространенность и факторы риска развития кишечной паразитизма среди двух коренных субэтнических групп в полуостровной Малайзии

Юии Тенг Чин, Ивонна Аи Лиан Лим, Чун Ви Чонг, Синди Шуан Ю. Дэ, Иван Кок Сенг Яп, Су Цзин Ли, Миан Зи Ти, Винни Вэй Инь Сиоу и Кек Хенг Чуа

Аннотация

Цель: Кишечные паразитарные инфекции (АПИ) среди коренных народов были широко подтверждённы (документально) в Малайзии, однако, распространенность этих инфекций остается на высоком уровне. В прошлом, большинство исследований были сосредоточены на конкретных видов паразитов, но полипаразитизм получил ограниченное внимание. Кроме того, эпидемиологические исследования по вопросам коренных народов, как правило, рассматривают их как однородную группу, тогда как в действительности различных субэтнических группы имеют разные культурные и жизненные практики. Изменения в образ жизни, такие как личные методы гигиены могут предрасполагать различные группы для различных паразитарных инфекций. Для лучшего понимания распространенности и факторов риска кишечной паразитизма среди различных субэтнических групп, настоящее исследование было проведено среди двух субэтнических групп коренных народов (Темуан и Мах Мери), проживающих в штате Селангор, Малайзии.

Методы: межгрупповые исследование, которые сосредоточены на двух различных субэтнических групп проводилось с февраля по сентябрь 2014 года. Фекальные образцы были собраны из 186 участников и

исследовали с помощью методом формалин-эфирного осаждения. Молекулярный подход был принят для проведения генетической характеристики паразитов. Кроме того, были использованы вопросники для получения информации о демографических, социально-экономических слоев и поведенческих рисков, связанных с участниками, а также информацию об их окружающих условиях. Статистический анализ (т.е. двоичную и многофакторный логистический регрессионный анализ) были проведены для оценки факторов риска.

Результаты: Для сообществ Темуан, трихоцефалез (64,2%) был самой распространенной инфекцией, предшествующий анкилостомоз (34%), аскаридоз (7,5%), лямблиоз (14,2%) и амебиаз (7,5%). Что касается общин Мах Мери, трихоцефалез (77,5%) преобладали над аскаридоз (21,3%), нематоды (15%), лямблиоз (7,5%) и амебиаз (3,8%). Наблюдались существенные различия в пропорциях Трихоцефалез, аскаридоз и анкилостомидозов инфекций между субэтнических групп Темуан и Мах Мери. Полипаразитизм было более распространено среди субэтнических группы Темуан (41,5%) по сравнению с суб-этнической группы Мах Мери (32,5%), причем большинство участников является переносчиком двух паразитов одновременно (Темуан: 33%, Мах Мери: 20%). Власоглав и человеческая аскарида были наиболее распространены (10%) среди общин Мах Мери, в то время как сочетанная инфекция T. Trichiura (Власоглав) нематодами (19,8%) был наиболее распространенным среди общин Темуан. Многофакторные анализы показали, что являясь безработными, имея большую семью и пить сырую воду оказалась быть в значительной степени связано с кишечным паразитизмом.

Заключение: Настоящее исследование выдвигает на первый план существенные полипаразитизм и факторы риска для инфекций в субэтнических групп Темуан и Мах Мери. Высокая распространенность АПИ среди этих двух субэтнических групп указывает на то, что паразитарные инфекции являются важными проблемами здравоохранения в этих общинах. Следовательно, крайне важно для реализации эффективных стратегий вмешательства, такие как периодический профилактической химиотерапии в сочетании с медико-санитарного просвещения в целях сокращения и искоренения этих инфекций.

Translated from English version into Russian by Turdimurot Rakhmonov, through

Prevalencia y factores de riesgo del parasitismo intestinal en dos grupos subétnicos indígenas en Malasia peninsular

Yuee Teng Chin, Yvonne Ai Lian Lim, Chun Wie Chong, Cindy Shuan Ju Teh, Ivan Kok Seng Yap, Soo Ching Lee, Mian Zi Tee, Vinnie Wei Yin Siow y Kek Heng Chua

Resumen

Generalidades: Las infecciones parasitarias intestinales (IPI) entre los pueblos indígenas han sido ampliamente documentadas en Malasia. Sin embargo, la prevalencia de estas infecciones sigue siendo alta. En el pasado, la mayoría de los estudios se centraban en especies de parásitos específicas, pero apenas se prestó atención al parasitismo múltiple. Además, los estudios epidemiológicos que se realizan en las comunidades indígenas suelen percibir a estas poblaciones como un grupo homogéneo, cuando en realidad los diferentes grupos subétnicos tienen

prácticas culturales y formas de vivir diferentes. Las diferencias en los hábitos de vida, tales como las prácticas de higiene personal, pueden predisponer a los diversos grupos a infecciones parasitarias distintas. A fin de comprender mejor la prevalencia y los factores de riesgo del parasitismo intestinal en los distintos grupos subétnicos, se llevó a cabo un estudio de dos comunidades indígenas (los Temuan y los Mah Meri), que habitan en el estado de Selangor en Malasia.

Métodos utilizados: En el período comprendido entre febrero y septiembre de 2014, se realizó un estudio transversal que se centró en dos grupos subétnicos diferenciados. Se recogieron muestras fecales de 186 participantes, que fueron examinadas mediante la técnica de sedimentación con formol y éter. Se adoptó un método molecular para llevar a cabo una caracterización genética de los parásitos. Por otra parte, se distribuyeron cuestionarios con el objeto de recoger información sobre aspectos demográficos, situación socioeconómica y conductas de riesgo de los participantes, así como información sobre su entorno. A fin de medir los factores de riesgo, se realizaron análisis estadísticos (esto es, análisis de regresión logística binarios y multivariados).

Resultados: Con respecto a las comunidades Temuan, se descubrió que la infección más común era por tricuriasis (64,2%), seguida de las infecciones por anquilostoma (34%), ascariasis (7,5%), giardiasis (14,2%) y amebiasis (7,5%). En lo que respecta a las comunidades Mah Meri, la infección más común era por tricuriasis (77,5%), que prevalecía sobre la ascariasis (21,3%), la infección por anquilostoma (15%), la giardiasis (7,5%) y la amebiasis (3,8%). Se observaron importantes diferencias en las proporciones de las infecciones por tricuriasis, ascariasis y anquilostoma entre los grupos subétnicos Temuan y Mah Meri. Los casos de parasitismo múltiple eran más comunes en el grupo subétnico Temuan (41,5%), que en el grupo Mah Meri (32,5%), y la mayoría de los participantes eran portadores de dos parásitos simultáneamente (Temuan: 33%, Mah Meri: 20%). La infección conjunta por *Trichuris trichiura* y *Ascaris lumbricoides* era más prevalente (10%) entre las comunidades Mah Meri, mientras que la infección conjunta por *T. trichiura* y anquilostoma (19,8%) era más común en las comunidades Temuan. Los análisis multivariados revelaron que entre los factores que se asociaban de manera significativa con el parasitismo intestinal se encontraban la falta de empleo, el hecho de pertenecer a una familia numerosa y beber agua sin hervir.

Conclusión: El presente estudio pone de relieve la importancia que reviste el parasitismo múltiple y los factores de riesgo de infección en los grupos subétnicos Temuan y Mah Meri. La alta prevalencia de las IPI en estos dos grupos subétnicos indica que las infecciones parasitarias constituyen un importante problema de salud en estas comunidades. Por tanto, es imperativo poner en marcha estrategias de intervención sólidas, como la quimioterapia preventiva periódica, que debería complementarse con programas de educación sanitaria a fin de reducir y erradicar estas infecciones.

Translated from English version into Spanish by Suzanne Assenat, through

TRANSLATORS
WITHOUT BORDERS
