# Supplementary material for: Prevalence and risk factors of intestinal parasitism among two indigenous sub-ethnic groups in Peninsular Malaysia
Source: Infect Dis Poverty. 2016 Jul 18;5:77. doi: 10.1186/s40249-016-0168-z (PMC4950084; doi:10.1186/s40249-016-0168-z)
Supplement: Additional file 2: Additional tables. Table S1. — General characteristics of indigenous communities who participated in this study. Table S2. Predictive power of the enter and forward selection logistic regression model. (PDF 175 kb) [file 40249_2016_168_MOESM2_ESM.pdf]

Table S1: General characteristics of indigenous communities who participated in this study

Variables	Total (N=186)		Temuan (N=106)		Mah Meri (N=80)	
	No. examined	Percentage (%)	No. examined	Percentage (%)	No. examined	Percentage (%)
**DEMOGRAPHIC DATA**						
Age group						
<15 years	63	33.9	43	40.6	20	25.0
≥15 years	123	66.1	63	59.4	60	75.0
Gender						
Male	79	42.5	43	40.6	36	45.0
Female	107	57.5	63	59.4	44	55.0
**SOCIOECONOMIC STATUS**						
Education attainment						
Formal education	152	81.7	82	77.4	70	87.5
Informal education	34	18.3	24	22.6	10	12.5
Employment						
Unemployed	131	70.4	76	71.7	55	68.8
Size of family						
≥5 members	120	64.5	73	68.9	47	58.8
Household income						
<RM750*	132	71.0	62	58.5	70	87.5
Source of water supply						
Untreated water source	47	25.3	30	28.3	17	21.3
Presence of toilet						
Yes	151	81.2	85	80.2	66	82.5
Presence of domestic animals at household						
Yes	113	60.8	69	65.1	44	55.0
**PERSONAL HYGIENE PRACTICES**						
Boiling water before drinking						
Yes	126	67.7	73	68.9	53	66.3
Wearing shoes outside house						
Yes	135	72.6	80	75.5	58	72.5

*1 RM = 0.25 USD

Table S2: Predictive power of the enter and forward selection logistic regression model

**Observed**		**Overall indigenous people enter selection model**	**Overall indigenous people forward selection model**	**Temuan sub-ethnic group enter selection model**	**Temuan sub-ethnic group forward selection model**	**Mah Meri sub-ethnic group enter selection model**	**Mah Meri sub-ethnic group forward selection model**
Presence of parasitic infection (%)	Yes	97.9	100.0	100.0	100.0	100.0	100.0
	No	19.5	0	0	0	0	0
Overall percentage (%)		80.6	78.0	76.4	76.4	80.0	80.0
